# Access to Adolescent Pregnancy Prevention Information and Services in Ghana: A Community-Based Case-Control Study

**DOI:** 10.3389/fpubh.2019.00382

**Published:** 2019-12-13

**Authors:** Bright Opoku Ahinkorah, John Elvis Hagan, Abdul-Aziz Seidu, Eugene Budu, Thomas Hormenu, Joseph Kwame Mintah, Francis Sambah, Thomas Schack

**Affiliations:** ^1^Faculty of Health, The Australian Centre for Public and Population Health Research (ACPPHR), University of Technology Sydney, Sydney, NSW, Australia; ^2^Department of Health, Physical Education and Recreation, University of Cape Coast, Cape Coast, Ghana; ^3^Neurocognition and Action-Biomechanics-Research Group, Faculty of Psychology and Sport Sciences, Bielefeld University, Bielefeld, Germany; ^4^Department of Population and Health, University of Cape Coast, Cape Coast, Ghana

**Keywords:** access, adolescent, pregnancy, sexuality, reproductive health, Ghana

## Abstract

**Background:** Pregnancy among girls 10–19 years remains a challenge that requires critical resolution all over the world. Despite this worrying sexual phenomenon, research pertaining to prevention information and related services in Sub-Saharan nations like Ghana is sparse. This study sought to determine the influence of access to pregnancy prevention information and services on adolescent pregnancy in the Komenda-Edina-Eguafo-Abrem Municipality in the Central Region of Ghana.

**Methods and Results:** Adopting a matched case-control research design with a 1:1 mapping, female adolescents aged between 15 and 19 years in the KEEA Municipality were selected using a facility based sampling technique. Results from both bivariate and multivariate analyses revealed that non-pregnant adolescents were about two times more likely to have access to pregnancy prevention information from health workers compared to pregnant adolescents [OR = 0.57, 95% CI = (0.33–0.96), *p* = 0.036]. Likewise, pregnant adolescents were five times more likely to have access to pregnancy prevention information from media compared to non-pregnant adolescents [OR = 5.44, 95% CI = (2.64–11.23), *p* = 0.000]. Additionally, non-pregnant adolescents were two times more likely to receive information on pregnancy prevention from school compared to pregnant adolescents [OR = 0.48, 95% CI = (0.28–0.81), *p* = 0.006].

**Conclusion:** Sexuality and reproductive health (SRH) programme organizers should target specific intervention programmes that focus on training health workers and/or other analogous staff to enhance their awareness, attitudes, and skills to more effectively meet with the specific needs of adolescents. Specific health workers training and redesign of health facilities to foster more adolescent user friendly working environment (e.g., extension in operational times, reduction in fees of SRH services, transforming physical design to promote privacy or confidentiality) ought to be encouraged. Different media outreach programmes should also combine other community level events [e.g., informative methods through schools (e.g., focus group discussions, participatory learning), assisting connections to health services, community information network (e.g., use of sirens)] to provide well–tailored advocacy that would help modify SRH and sociocultural norms that hinder positive sexual behaviors among young people.

## Introduction

Adolescence is often noted as a transitional period characterized by good health. Despite this notion, adolescents (aged 10–19 years) are saddled with health risk exposures related to their sexuality and reproduction (1). For instance, adolescent pregnancy (AP) and its associated health and social implications signify a major public health concern that requires critical resolution in many nations (2, 3). Available statistics show that 11% of all deliveries and 14% of maternal losses globally are between 15- and 19-year-old females, with 95% of adolescent childbirths occurring in developing nations (4–6). Notably, adolescents' unwanted pregnancies are estimated at 7.4 million each year and 3 million unintended pregnancies and unsafe abortions are recorded among girls (7, 8). Almost 95% of APs happen in low- and middle-income countries (LMICs), where 36.4 million females become mothers before age 18 (3, 9). Estimates on young adolescents further reveal that 1 million births happen across young females aged 12–15 years every year (10).

Significantly, there seems to be some regional variations. For example, childbirths to adolescents as a percentage of all deliveries range from ~2% in China, to 18% in Latin America and the Caribbean, to more than 50% in Sub-Saharan Africa (11). Pregnancies among single young mothers are more likely to be unplanned, culminating in induced abortion and coerced sex that accounts for unintended APs among other negative implications (3, 11). Sub-Saharan Africa has recorded the highest occurrence of AP globally, with childbirths to adolescent mothers accounting for more than half of all births in the sub-region (3). Most of the countries with AP records above 30% happen in sub-Saharan Africa (12). Within sub-Saharan Africa, the proportion of AP ranges from 0.3% in Rwanda to 12.2% in Mozambique (11). A substantial proportion of these APs is wanted and may even be planned (13).

Ghana's AP situation is not dissimilar to other parts of sub-Saharan Africa and the rest of the world. AP is endemic in Ghana as there have not been significant changes in the rates between 2003 (14%) and 2008 (13%). About one in ten young females, between 15 and 19 years of age had begun childbearing in the cities whereas about twice this figure occur in the rural settings (14). Ghana continues to record higher rates of AP. Recent national report shows that 11% of adolescents aged 15–19 years had had a live birth, of which 3% are pregnant with first child and 14% had begun childbearing (15). The percentage of teenagers who had begun childbearing rose rapidly with age, from 1% at age 15 to 31% at age 19. Adolescents living in rural areas (17%), those residing in the Brong Ahafo, Central, and Volta regions (21–22%), those with no education (23%) and those in the second wealth quintile (21%) have the tendency to start childbearing earlier than other adolescents in Ghana (15). This national document stated that the number of young girls who got pregnant with a child in the Central Region stood at 7%. Available statistics for Central Region suggest that the Komenda-Edina-Eguafo-Abrem (KEEA) Municipality is one of the districts noted for the high prevalence of APs. The total APs in the Municipality is estimated at 17.5% (16, 17).

There are several detrimental health, psychosocial, and economic consequences connected with AP that are disturbing and should not be underestimated (18–21). Research till date has proven that pregnant females younger than 17 years have a higher incidence of health complications related to mother and child compared to grown-up women, though these complications may be more severe among youngest adolescents (22, 23). Generally, AP carries a greater risk of critical maternal and neonatal outcomes. For example, AP has been connected to medical problems, including but not limited to undesirable maternal weight increase, prematurity (birth at <37 weeks' gestation), pregnancy-induced hypertension, anemia, urinary tract infection, post-partum hemorrhage, mental disorders (e.g., depression), and STDs (3, 18, 22, 23). Available records show that nearly 14% of babies born to adolescents 17 years or younger are preterm compared to 6% for women 25–29 years of age. Young mothers who are 14 years and younger are more likely to give birth to underweight infants compared to other age groups, with adolescents in developing countries more vulnerable (11, 24–29). The psychosocial implications of AP include school dropout [with subsequent lower educational accomplishment, and reduced social prospects, including decrease lifetime earnings (abject poverty)], restricted vocational opportunities, parting from the child's father, divorce or separation, and in some settings, possible discrimination and denial by family or community members, violence, including suicide and homicide (30, 31). According to Upchurch and McCarthy (32), once pregnancy interferes with an adolescent's education, a low academic performance is usually recorded.

Due to these AP challenges, various strategies have been adopted to deal with them. One of the key strategies in addressing adolescent pregnancy which form a major aspect of adolescent sexual and reproductive health service delivery is access to pregnancy prevention information and services (33–35). Access to pregnancy prevention information and services includes but not limited to information regarding anatomy and the physiology of biological sex and reproduction, healthy sexual development, gender identity, interpersonal relationships, affection, sexual development, intimacy, body image for all adolescents (33, 36), and contraceptives (37–39). Such information and services are provided by teachers, health workers and parents have major responsibilities toward the sexual and reproductive health of adolescents (40–42).

Despite the importance of teachers, health workers and parents in providing such information and services to adolescent girls, negative socio-cultural norms regarding sexuality education may hinder the role such stakeholders play in the lives of adolescent girls (16, 43, 44). Parents usually feel uncomfortable discussing sexuality and reproductive health issues with their own children due negative norms in society (43, 45). Similarly, de Paul Kanwetuu et al. (46) asserted that in Ghana, many teachers and parents fear that teaching adolescents' sexuality education will enable adolescents engage in sex earlier and more often than anticipated, hence prefer keeping adolescents on the dark side of sexuality education. Some community members in Ghana have also blamed teachers and health care workers for corrupting children by teaching about sex and providing contraceptive methods (45). As a result of this criticism, some adolescent girls rather obtain such information and services from friends and the media (43) although these sources have been found to be ineffective (44, 47, 48).

Considerable efforts from stakeholders should now target not only general health service delivery but also make conscious attempt to provide adolescent friendly networks that are available, acceptable, impartial, appropriate, and operational in promoting sexuality and reproductive health. These concerted efforts should aim to facilitate the capacity and preparedness to find sexuality and reproductive information and services, predominantly among adolescents who need them the most (1, 49). Therefore, investigating whether pregnancy prevention information and services through an empirical enquiry could help change attitudes and influence the intentions to practice safe sexual behavior amongst adolescents in the KEEA Municipality would be crucial. This current study seeks to explore the various sources of pregnancy prevention information and services among adolescent girls in one of the widespread areas (KEEA Municipality) of the Central Region of Ghana and determine whether these sources are protective or risk factors for adolescent pregnancy.

## Materials and Methods

### Study Design

The study design was a matched case-control research design with a 1:1 mapping. Within this study, a case was referred to any young female (aged 15–19 years) who was pregnant at the time of the study and sought medical care in any of the health centers in the KEEA Municipality. Controls were females between 15 and 19 years (during the data collection period) who have never experience pregnancy. The choice of this research approach is based on the justification that it gives researchers the ability to find risk factors connected with health behaviors such as adolescent pregnancy by exclusively concentrating on cases (adolescent group with pregnancy) and the controls (adolescent group without pregnancy) and afterwards identify the exposure comparable to studied variables among subjects in each group retrospectively.

### Study Area

The study setting was the KEEA Municipality. According to the 2010 Population and Housing Census, the KEEA Municipality's population stood at 144,705. The inhabitants constituted 6.6% of the region's total residents. Males constitute 48.2% of the total population whereas females represent 51.8%. Sixty-four percent (64%) of the population is denoted as rural. The district's population is considered young with about 40% of the people below 15 years, showing an expansive population pyramid which decreases with a small number of aging people (8.6%). The General Fertility Rate of 105.0 births per 1,000 females aged 15–49 years and a Total Fertility Rate of 3.6 are recorded within the Municipality. The Crude Birth Rate (CBR) also stands at 24.6 per 1,000 population (50). Public healthcare delivery services within the Municipality are organized by the Municipal Health Management Team (MHMT). This management team offers support to sub-district on disease prevention and control, health promotion, and general public health education. Majority of the health centers are situated within the Elmina geographical environs (i.e., the Municipal capital). Due to the relatively vast land mass of the Municipality, access to health facilities is a major problem (50).

### Population and Sampling

The study population included female adolescents (15–19) years in the KEEA Municipality. A sum of 7,667 females (10–19 years), including both pregnant and non-pregnant adolescents represented the study population (50). Out of the population of 7,667, a sample size of 400 was generated with Krejcie and Morgan (51) table of determining sample sizes. Facility based sampling method was used to select 200 pregnant and 200 non-pregnant adolescents for the study. This sampling procedure involves engaging participants of the target population from diverse facilities such as correctional and drug treatment centers, sexually transmitted diseases clinics or general health centers, and hospitals (52). Using this sampling procedure, pregnant adolescents using antenatal services from five health facilities within the Municipality were purposively and conveniently chosen earlier. The selected health facilities within the Municipality are the Kissi health center, Komenda health center, Elmina urban health center, Agona health center, and Ankaful General Hospital. From each of these five health facilities, 40 pregnant adolescents and 40 non-pregnant adolescents were selected. Thus, 80 research participants were selected from each of the five health facilities. We conveniently matched each pregnant adolescent picked with a non-pregnant adolescent within the same age group from the five selected health facilities. The sampled pregnant adolescents and non-pregnant adolescents were used as the cases and controls for the current study. A facility-based sampling procedure was used because reaching potential study participants through other sampling techniques would have been extremely difficult.

### Instrumentation

The data collection instrument for this study was a questionnaire. This study did not use an existing questionnaire but the questionnaire was developed by the researchers from scratch. The items on the questionnaire were generated from the findings of previous studies related to the current study which were carried out in other geographical settings (37, 38, 40, 42, 53). The questionnaire had introductory data on participants' demographics (e.g., religion, mother, and father's educational status, employment status). Other listed items on the questionnaire centered on access to pregnancy prevention information and services, with participants expected to provide answers on a binary scale (e.g., Yes or No). Some item specific examples on the questionnaire were “discussion of issues on pregnancy prevention with parents,” “access to information on pregnancy prevention from health workers” and access to information on pregnancy prevention from media.” To ensure validity of the questionnaire, the questionnaire was presented to three expert professors in adolescent health education and promotion for their comments. The inputs were to guarantee that each item formulated and whole content of the questionnaire reflected the central focus of the study (54). A pre-tested was then conducted on the questionnaire using 50 teenagers in the Cape Coast Metropolis. The instrument produced a reliability coefficient of 0.75 with the Kuder-Richardson formula 21 (KR-21), a figure that is considered satisfactory (55).

### Data Collection Procedure

The data collection process began with establishing a preliminary rapport with the health directors of the five (5) selected health facilities from where the data collection took place. With the help of two researchers and two trained assistants, data collection from the respondents in their respective health facilities lasted 12 weeks. The duration for the data collection was agreed upon with the staff of the health centers to ensure that their daily job related services were not disrupted. For each visit for data collection, the central aim of the study was vividly described to the respondents. After administering a questionnaire to each pregnant adolescent, a non-pregnant counterpart was also identified to complete one of the data collection instruments at each health center during the entire period. The administration of the questionnaire was completed concurrently in the meeting rooms of the selected health facilities. Prior to the completion of the questionnaire, the standard instructions for usage were always read to all participants who had accepted to give answers to the questions on the instrument. The trained research assistants provided support with the completion of the questionnaire to some respondents, especially to study participants who wanted help with the understanding of some information in the local dialect because they lack proficiency in English language. This was possible because the trained research assistants understood very well both English and the local dialect of the research participants and were also trained on how to translate the items on the questionnaire from English to the local dialect of the research participants. The data collection procedure began after informing participants about the rationale of the study and respondents consenting to partake in the study. The respondents were then assured that information collected or gathered was merely for educational reasons. The decision to participate was completely voluntary and that participants could withdraw from the study at any time they felt uncomfortable. Other ethical issues observed in the study were anonymity, respect, and confidentiality, and that these guidelines were maintained during the entire data collection process by avoiding any personal identifier from the research instrument.

### Data Analysis

After data collection, the researchers checked through all the questionnaires. Each filled-in questionnaire was subsequently coded in coding booklet by the researchers to reduce inaccuracies. Data were thoroughly cleaned and entered into SPSS version 21 for analysis. Both bivariate and multivariate analyses were performed. For the bivariate analysis, Pearson's chi-square test of independence between the dependent variable (pregnancy status) and each of the independent variables (access to pregnancy prevention information and services) to determine any statistically significant association between the set of variables was employed. The independent variables that showed statistical significance with the dependent variable were further entered into a multiple regression model to determine their impact on the dependent variable. Odds ratio (OR) at 95% confidence intervals (CI) and *p*-values were derived for all significant variables in the bivariate analysis. Variables identified to be significant at the bivariate level (*p* ≤ 0.05) were entered into a multivariate analysis model in order to control for the effect of confounding variables. All odds ratios <1 were reversed to whole numbers and interpretations given based on the reverse values to make understanding easy.

## Results

### Socio-Demographic Characteristics of Respondents

Table 1 results indicate that in relation to religion, most of the pregnant and non-pregnant adolescents were Christians (95 and 94.5%). With respect to educational status, majority of the pregnant and non-pregnant adolescents had their education beyond primary level (64 and 52%), respectively. Regarding mothers' educational level, 61.2 and 85.7% of the pregnant and non-pregnant adolescents' mothers had either primary educational status or beyond. Again, on fathers' educational status, greater proportions of pregnant adolescents' fathers (70.1%) and their non-pregnant adolescents' fathers (89.3%) had either primary education or more. Majority of the pregnant and non-pregnant adolescents' mothers (77.5% and 88.4%) had employment. Similarly, 77.5% of the pregnant adolescents' fathers and 89.8% of non-pregnant adolescents' fathers were also employed.

**Table 1:** Socio-demographic characteristics of respondents.

**Socio-demographic factors**	**Pregnant (*****n*** **=** **200)**		**Non-pregnant (*****n*** **=** **200)**	
	***n***	**%**	***N***	**%**
**Religion**				
Non-Christians	10	5	11	5.5
Christians	190	95	189	94.5
**Educational level**				
Primary education or less	5	2.5	2	1
> primary education	195	97.5	198	99
**Mother's level of education**				
Primary education or less	59	38.8	24	14.3
> primary education	93	61.2	144	85.7
**Father's level of education**				
Primary education or less	40	29.9	16	10.7
> primary education	94	70.1	133	89.3
**Mother's employment status**				
Unemployed	43	22.5	23	11.6
Employed	148	77.5	176	88.4
**Father's employment status**				
Unemployed	42	24.3	20	10.2
Employed	131	75.7	177	89.8

### Bivariate Analysis on Pregnancy Prevention Information and Services, and Adolescent Pregnancy Status

The results of a bivariate analysis of the relationship between access to pregnancy prevention information and services, and adolescent pregnancy in the KEEA Municipality are presented in Table 2. All the independent variables were statistically significant with adolescent pregnancy within the bivariate analysis. Regarding access to pregnancy prevention information from parents, 72% of the pregnant adolescent did not discuss issues on pregnancy prevention with parents. Again, 64% of adolescent girls who discuss pregnancy prevention issues with friends were not pregnant. Furthermore, almost 81% of non-pregnant adolescent girls receive information on pregnancy prevention from health workers. Ninety-one percent of pregnant adolescent girls obtained information on pregnancy prevention from media whiles ~89% of pregnant adolescents did not have access to pregnancy prevention services from health facilities. Similarly, 68% of the non-pregnant adolescents received information on pregnancy prevention from school. From the chi-square analysis, discussion of issues on pregnancy prevention with parents [χ^2^_(1)_ = 52.2, *p* < 0.05], discussion of pregnancy prevention issues with friends [χ^2^_(1)_ = 9.2, *p* < 0.05], access to information on pregnancy prevention from health workers [χ^2^_(1)_ = 19.2, *p* < 0.05], access to information on pregnancy prevention from media [χ^2^_(1)_ = 12.1, *p* < 0.05], access to pregnancy prevention services from health facilities [χ^2^_(1)_ = 9.3, *p* < 0.05], and access to pregnancy prevention information from school [χ^2^_(1)_ = 18.0, *p* < 0.05] revealed a statistically significant association with pregnancy status.

**Table 2:** Bivariate analysis on access to pregnancy prevention information and services and adolescent pregnancy status.

	**Pregnant *n* (%)**	**Non-pregnant *n* (%)**	**Chi-square (*χ^2^*)**	***P*-value**
**Discuss issues on pregnancy prevention with parents**				
No	144 (72)	72 (36)		
Yes	56 (28)	128 (64)	52.2	0.000^*^
**Discuss pregnancy prevention issues with friends**				
No	98 49)	72 (36)		
Yes	102 (51)	128 (64)	9.2	0.002^*^
**Receive information on pregnancy prevention from health workers**				
No	79 (39.5)	39 (19.5)		
Yes	121 (60.5)	161 (80.5)	19.2	0.000^*^
**Obtain information on pregnancy prevention from media**				
No	18 (9)	43 (21.5)		
Yes	182 (91)	157 (78.5)	12.1	0.001^*^
**Access to pregnancy prevention services from health facilities**				
No	177 (88.5)	154 (77)		
Yes	23 (11.5)	46 (23)	9.3	0.002^*^
**Receive information on how to prevent pregnancy from school**				
No	106(53)	64 (32)	18.0	0.000^*^
Yes	94(47)	136 (68)		

* *Significant results*.

### Multivariate Analysis on the Influence of Access to Pregnancy Prevention Information and Services on Adolescent Pregnancy Status

The results of the multivariate logistic regression analysis on access to pregnancy prevention information and services on adolescent pregnancy in KEEA Municipality are presented in Table 3. Five independent variables (i.e., discussion of issues on pregnancy prevention with parents, access to pregnancy prevention information from health workers, access to pregnancy prevention information from media, access to pregnancy prevention services from health facilities, and access to pregnancy prevention information from school) showed statistically significant association with adolescent pregnancy. The full model with all the predictors was statistically significant [χ^2^ (5, *n* = 400) = 94.6, *p* < 0.05], demonstrating that the model was able to discriminate between female adolescents who were pregnant and those who were not pregnant. The entire model explained between 21% (Cox & Snell *R*^2^) and 28% (Nagelkerke *R*^2^) of the variance in pregnancy status. Specifically, non-pregnant adolescent girls were three times more likely to have access to pregnancy prevention information from parents than their pregnant adolescent counterparts [OR = 0.30, 95% CI = (0.19–0.48), *p* = 0.000]. Similarly, non-pregnant adolescent girls were about two times more likely to have access to pregnancy prevention information from health workers than their pregnant adolescent counterparts [OR = 0.57, 95% CI = (0.33–0.96), *p* = 0.036]. Further, pregnant adolescent females were five times more likely to have access to pregnancy prevention information from media than their non-pregnant adolescent counterparts [OR = 5.44, 95% CI = (2.64–11.23), *p* = 0.000]. Non-pregnant adolescent females were about two times more likely of having access to pregnancy prevention services from health facilities than their pregnant adolescent colleagues [OR = 0.52, 95% CI = (0.28–0.95), *p* = 0.033]. Compared to pregnant adolescents, non-pregnant adolescent counterparts were two times more likely to receive information on pregnancy prevention from school [OR = 0.48, 95% CI = (0.28–0.81), *p* = 0.006].

**Table 3:** Influence of access to pregnancy prevention information and services on adolescent pregnancy.

	**Pregnant %**	**Non-pregnant %**	**B**	**Wald**	**OR (CI)**	***p*-value**
Pseudo *R*^2^	0.21–0.28					
*χ^2^*	94.6					
*p*-value	0.000					
**Discuss issues on pregnancy prevention with parents**						
No	144 (72)	72 (36)	−1.21	25.58	Ref	
Yes	56 (28)	128 (64)			0.30 (0.19–0.48)	0.000^*^
**Discuss pregnancy prevention issues with friends**						
No	98 49)	72 (36)	−0.40	2.69	Ref	
Yes	102 (51)	128 (64)			0.67 (0.42–1.08)	0.101
**Receive information on pregnancy prevention from health workers**						
No	79 (39.5)	42 (21)	−0.57	4.41	Ref	
Yes	121 (60.5)	158 (79)			0.57 (0.33–0.96)	0.036^*^
**Obtain information on pregnancy prevention from media**						
No	18 (9)	43 (21.5)	1.69	21.06	Ref	
Yes	182 (91)	157 (78.5)			5.44 (2.64–11.23)	0.000^*^
**Access to pregnancy prevention services from health facilities**						
No	177 (88.5)	154 (77)	−0.66	4.52	Ref	
Yes	23 (11.5)	46 (23)			0.52 (0.28–0.95)	0.033^*^
**Receive information on pregnancy prevention from school**						
No	106(53)	64 (32)	−0.74	7.52	Ref	
Yes	94(47)	136(68)			0.48 (0.28–0.81)	0.006^*^

* *Significant results*.

## Discussion

This study sought to assess the relative influence of how access to pregnancy prevention information and services could impact on adolescent pregnancy status in the KEEA Municipality of Ghana. The results indicate that non-pregnant adolescents were more likely to have access to pregnancy prevention information from parents compared to pregnant adolescents, a finding that corroborates previous studies (56–59). Therefore, sociocultural norms surrounding sexuality and reproductive health issues (SRH) in typical Ghanaian and other sub-Saharan homes should not be underestimated. The culture of silence or openness (i.e., obtaining information, discussions, and expressions) between parents and their adolescent girls on sexuality issues can inversely be connected to the onset of sexual debut and subsequent negative implications (e.g., pregnancy, abortions) and vice versa (16, 43). Within local set-ups, many adolescent girls have few opportunities to express themselves without prejudice, trust people and places to solicit for sexual information and support. The absence of anonymity, confidentiality, and environment free from judgement are obstacles to adolescent girls seeking for SRH information, learn practical skills, and receive support in articulating their complaints related to their personal lives and SRH issues (60).

Research evidence through intervention studies indicates the possibility to enhance the structure and/or content of deliberations, and to create awareness and challenge sociocultural norms that hamper discussions on sexuality among adolescents (61). For instance, a parent-approach intervention programme to support seventh graders' families' capacities to interact with their teenagers, offer help, practice positive parenting, and promote their participation was investigated among low-income Latino homes in Miami. After 36 months, study participants in the treatment groups were less likely to have an STI and unprotected sex at last sexual encounter comparable to their counterparts in the two control conditions (62). This finding underscores the need to improve adolescent-parent communication and information sharing (63). This communication approach helps adolescents to establish individual values toward healthy sexual behaviors such as abstinence, improved contraception, how to prevent HIV, and other sexually transmitted diseases. Despite the significant role parents and the extended family might have toward SRH awareness and growth of young people (49, 64), research from sub-Saharan Africa and other geographical locations' shows that interactions between adolescents and their parents on matters related to sexual affairs and early pregnancy is sparse (61, 65, 66). More studies are required to investigate obstacles to communication about sexuality such as lack of parental awareness, dependence on school teachers, and a perception that speaking about sexuality promotes sex (61).

Not different from previous studies [e.g., Maravilla et al. (53)], non-pregnant adolescents in the current study were more likely to have access to pregnancy prevention information from health facilities and health workers compared to pregnant adolescents. Available literature has shown that health workers are effective in the provision of community health information to both young and old people on SRH issues to develop not only adolescent reproductive health but also a wider range of maternal and child health issues (67–69). Similarly, although studies have shown that adolescents feel ashamed going to these health centers for information or services relating to sex and contraception due the perception of unwelcoming attitude at the facility (70, 71), there is evidence that delivering quality services that are tailored to adolescents needs (e.g., extension in operation hours, reduction in prices of SRH services, transforming physical design to promote privacy or confidentiality) may result in an improvement in service use, adherence to contraceptive methods, and increase the probability of seeking on-going health care (1, 72). For example, an investigation in Uganda on the influence of health center reorganization at different stages of health worker training and empowering of health teams' ability for training and supervision involving adolescents revealed more than double upsurge in self-reported usage of health services such as family planning and STI services (73). Mbonye reported a more modest rise in self-reported use of family planning services among adolescents with intervention compared with the control communities. Some studies in Ghana have shown that there is improvement with the introduction of youth-friendly services in the country (74, 75). Given that most SRH services are often provided at the premises of health facilities (e.g., clinics, hospitals, centers), these SRH health services can also be delivered at notably gatherings within the community where adolescents live and converge. Possible places for outreach services may include but not limited to schools, workplace, streets, malls, homes, youth centers, pharmacies, entertainment grounds (e.g., during festivals), and storefronts (1). This outreach approach can help contribute to the less risk of APs despite on-going sociocultural barriers.

Another finding from the current study indicates that non-pregnant adolescents were more likely to have access to pregnancy prevention information from media compared to pregnant adolescents. In line with previous studies [e.g., (47, 76, 77)] on SRH, the media offers a remarkable role in the lives of adolescents by giving them enough information on their sexuality. The mass media serves as one of the great tools for the youth in terms of accessibility to information during this contemporary era (78). The media encompasses varied means by which a greater audience receive information and entertainment (79). One key element of the media is the capacity to circulate materials on a broader perspective, the convenience, and the privacy with which adolescents can search for any information on this media such as the internet (80) are in calculable. Therefore, the media and other communication networks [i.e., print (e.g., newsletters, magazines, flyers) and electronic (e.g., internet; social media- snapchat, twitter, whatsapp, instagram), documentaries] can provide awareness and motivating discussions about SRH issues. Lou et al. (81) explained that adolescents and young adults with a keen romantic and sexual interest during their reproductive development, who have access to the various forms of media, tend to use them are relatively assured of privacy, safety, and confidentiality in learning about SRH issues, including pregnancy. The media could be more effective and a powerful tool in societies (e.g., sub-Saharan Africa) where speaking about sexual issues with adolescents and young adults is still seen as a source of worry for many educators and parents (43, 81). Hence, the media could be used as a kind of sexual “super peer” for adolescents and young adults looking for information on their sexuality (82). Future studies through intervention and longitudinal designs could target which media strategies could best promote SRH issues among adolescents.

The current study also revealed that non-pregnant adolescents were more likely to receive information on pregnancy prevention from schools compared to pregnant adolescents. Research evidence indicates that schooling has continually been identified to be related to a wide range of SRH behavioral tendencies such as contraceptive use, age of marriage, number of births, and use of health services (1). A systematic review on risk and protective causes of SRH in low- and middle-income countries found that current in-school adolescents are less likely ever to have had a sexual experience compared to individuals who quit school early. Additionally, the longer time (i.e., in years) adolescents spend in educational institutions, the more likelihood that current contraceptive methods would be utilized. A different review from Eastern, Southern, and Central Africa established that secondary education was inversely related to HIV rates and decrease risky sexual behaviors [e.g., early sexual debut, number of sexual/casual partners, and unprotected sex (83–86)]. Muhwezi et al. (44) reiterated that if adolescents are permitted early and given unrestricted access to SRH education, these young people are more likely to take less risk toward initiating sexual activity. Therefore, education empowers young girls in their sexual relations and practice safer sex (87).

## Limitations

Despite the relatively large sample size of the current study, the noted findings have some limitations. First, only five health facilities in the KEEA Municipality were used for the study, thus restrict generalizations it to other settings. Secondly, causal relationships could not be drawn in the current study because of using a case-control design characterized by sampling errors, and recall bias or memory loss. These shortcomings were minimized by sampling cases and controls that have comparable attributes beside pregnancy status. Again, participants were allowed considerable time to recollect various events that had happened previously. The field assistants who assisted in the data collection activities were also given adequate training prior to the data collection exercise. We do also recognize that there could be potential variations between pregnant and non-pregnant adolescents across their socio-demographic characteristics. However, since the rationale of the study was on access to pregnancy prevention information and services, the parameters of significance for the socio-demographic characteristics of the two groups were not considered, and so may affect our current findings. Further, the use of questionnaire may have restricted adolescents on their expression on key matters or may have misunderstood some of the questions. Hence, questionnaire usage limits the detection of all misinterpretations despite the presence of a researcher and trained assistants. Questions on delicate issues like pregnancy may be prone to social desirability issues such as recall biases, memory disturbance, and under or over reporting of a measure commonly inherent with self-reported measures.

## Practical Implications

Several opportunities exist for improving adolescents' SRH issues from different perspectives. Barriers to communication about SRH issues have been inhibiting adolescents, especially girls from inquiring information, deliberating, and articulating their reservations on SRH matters, mostly with reference to communicating with parents, other adults in the family and/ or neighborhoods/communities. Most adolescent girls have few platforms to express themselves without prejudgment or stereotypes, aware of few people and areas to seek SRH information and help. Consequently, specific programmes that create a physical environment (i.e., avenues/platforms) where young females can converge frequently; offer help to adolescents via a matured or peer mentor-mentee interactions; and provide life skills (e.g., persuasive communication and negotiation skills, literacy training) and/or vocational skills training (e.g., ICT, entrepreneurial skills) alongside with socialization and recreation can help mitigate these barriers. Parents could be given support services through advocacy that help develop parental responsiveness and openness to sociocultural norms that hinders parent-child connectedness on matters of SRH would be worthwhile. With respect to health facility services, providers could guarantee access to excellent adolescent user-friendly and comprehensive services delivered by healthcare personnel that are skilled to work with these young girls. These healthcare personnel ought to be professionally prepared to deliver precise, well-balanced sexual and reproductive health education as well as information on contraception and condom usage. Adolescents would then have the means to adopt protective measures against unplanned pregnancies themselves, provided within a context of optimal SRH, without stigmatization or prejudice. Varied media approaches and programmes ought to combine other community level events (e.g., the introduction of other informative resources through schools, assisting connections to health services, community information network through the use of sirens) to provide well–tailored advocacy that would help modify SRH and sociocultural norms that hinder positive sexual behaviors among young people. Another area of importance is strengthening curriculum and teacher skills necessary for improving SRH. To enable adolescents to personalize what they learn and future use, SRH issues must be facilitated through diverse and interactive methods (e.g., role play, demonstrations, and focus group discussions). These approaches should include cognitive learning through personal reflections and critical thinking on matters such as gender norms and practicing with new practical skills (e.g., persuasive communication). Hence, the classroom climate in schools should help adolescents improve their cognitive skills that encourages and also prompts adolescents' to interrogate their normative sociocultural practices as well as behaviors that challenge their health and well-being.

## Conclusion

There are numerous health, sociocultural challenges facing adolescents in developing countries like Ghana. Current findings suggest that communication related issues on pregnancy prevention with parents, access to pregnancy prevention information from health workers, access to pregnancy prevention information from media, access to pregnancy prevention services from health facilities, and access to pregnancy prevention information from school are associated with adolescents' pregnancy in the KEEA municipality. Major stakeholders (e.g., parents, local health directorates, school directorates, community leaders, and media houses) should help provide a supportive environment for adolescents SRH through local advocacy and policy redevelopment as well as technical support for training on SRH matters. This developmental approach should boost institutional collaborations [e.g., health institutions, schools, media houses, and other facilitators or service providers (e.g., health workers, teachers, and journalists)] through planning, implementing, monitoring, and evaluating multi-sectoral SRH programmes. Such programmes on SRH should target specific interventions that focus on training health workers and/or other analogous facilitators to enhance their awareness, attitudes, and skills to more effectively meet to the specific needs of adolescents. For example, specific health workers and other facilitators' training within their institutional setup to make delivery services more adolescent user friendly (e.g., extension in operational time, reduction in the cost of SRH services, transforming physical design to promote privacy, or confidentiality) should be encouraged. Other SRH training programmes could also target out-of-facility and/or institutionalized interventions (e.g., peer focus group discussions, participatory learning, and mentor-mentee groups) for the broader adolescent population within communities through the dissemination of information. More parental-adolescent connectedness through persuasive communication about their children's sexuality should be encouraged. These avenues may help build knowledge on pregnancy risk, life skills (e.g., communication and coping skills as well as stimulate critical reflections) on sexuality education. Future research should help identify barriers or obstacles that inhibit access to pregnancy and other sexuality information among adolescents with a larger sample across different districts in Ghana.

## Data Availability Statement

The datasets generated for this study are available on request to the corresponding author.

## Ethics Statement

The studies involving human participants were reviewed and approved by Research and Ethics Committee of University of Cape Coast, Ghana (UCCIRB/CES/2016/04) and the Ghana Health Service Ethics Review Committee (GHS-ERC: 13/12/2016). Written informed consent to participate in this study was provided by the participants' legal guardian/next of kin. Authorization to undertake the current study was also sought from the Central Regional Health Directorate, the KEEA Municipality Health Directorate, and the health facilities used for this research.

## Author Contributions

BA conceived the study. BA, A-AS, and JH designed and performed the analysis and the write up on data and methods. BA, A-AS, FS, TH, EB, and JH designed the first draft of the manuscript. BA, JH, A-AS, EB, FS, TH, JM, and TS revised and proof read the manuscript for intellectual content and gave consent for the final version to be published.

### Conflict of Interest

The authors declare that the research was conducted in the absence of any commercial or financial relationships that could be construed as a potential conflict of interest.
